# Non-small cell lung cancer and the tumor microenvironment: making headway from targeted therapies to advanced immunotherapy

**DOI:** 10.3389/fimmu.2025.1515748

**Published:** 2025-02-10

**Authors:** Anna De Lucia, Lucia Mazzotti, Anna Gaimari, Matteo Zurlo, Roberta Maltoni, Claudio Cerchione, Sara Bravaccini, Angelo Delmonte, Lucio Crinò, Patricia Borges de Souza, Luigi Pasini, Fabio Nicolini, Fabrizio Bianchi, Manel Juan, Hugo Calderon, Chiara Magnoni, Luca Gazzola, Paola Ulivi, Massimiliano Mazza

**Affiliations:** ^1^ Advanced Cellular Therapies and Rare Tumors Unit, IRCCS Istituto Romagnolo per lo Studio dei Tumori (IRST) “Dino Amadori”, Meldola, Italy; ^2^ Department of Biomedical and Neuromotor Sciences, University of Bologna, Bologna, Italy; ^3^ Healthcare Administration, IRCCS Istituto Romagnolo per lo Studio dei Tumori (IRST) “Dino Amadori”, Meldola, Italy; ^4^ Hematology Unit, IRCCS Istituto Romagnolo per lo Studio dei Tumori (IRST) “Dino Amadori”, Meldola, Italy; ^5^ Department of Medicine and Surgery, “Kore” University of Enna, Enna, Italy; ^6^ Medical Oncology Department, IRCCS Istituto Romagnolo per lo Studio dei Tumori (IRST) “Dino Amadori”, Meldola, Italy; ^7^ Unit of Cancer Biomarker, Fondazione IRCCS Casa Sollievo Della Sofferenza, San Giovanni Rotondo, FG, Italy; ^8^ Department of Immunology, Institut D’Investigacions Biomèdiques August Pi i Sunyer (IDIBAPS), Barcelona, Spain; ^9^ Translational Oncology Unit, IRCCS Istituto Romagnolo per lo Studio dei Tumori (IRST) “Dino Amadori”, Meldola, Italy

**Keywords:** non-small cell lung cancer (NSCLC), tumor microenvironment (TME), tyrosine kinase inhibitors (TKIs), immune checkpoint inhibitors (ICIs), chimeric antigen receptor (CAR) T cell therapy

## Abstract

Over the past decades, significant progress has been made in the understanding of non-small cell lung cancer (NSCLC) biology and tumor progression mechanisms, resulting in the development of novel strategies for early detection and wide-ranging care approaches. Since their introduction, over 20 years ago, targeted therapies with tyrosine kinase inhibitors (TKIs) have revolutionized the treatment landscape for NSCLC. Nowadays, targeted therapies remain the gold standard for many patients, but still they suffer from many adverse effects, including unexpected toxicity and intrinsic acquired resistance mutations, which lead to relapse. The adoption of immune checkpoint inhibitors (ICIs) in 2015, has offered exceptional survival benefits for patients without targetable alterations. Despite this notable progress, challenges remain, as not all patients respond favorably to ICIs, and resistance to therapy can develop over time. A crucial factor influencing clinical response to immunotherapy is the tumor microenvironment (TME). The TME is pivotal in orchestrating the interactions between neoplastic cells and the immune system, influencing tumor growth and treatment outcomes. In this review, we discuss how the understanding of this intricate relationship is crucial for the success of immunotherapy and survey the current state of immunotherapy intervention, with a focus on forthcoming and promising chimeric antigen receptor (CAR) T cell therapies in NSCLC. The TME sets major obstacles for CAR-T therapies, creating conditions that suppress the immune response, inducing T cell exhaustion. To enhance treatment efficacy, specific efforts associated with CAR-T cell therapy in NSCLC, should definitely focus TME-related immunosuppression and antigen escape mechanisms, by combining CAR-T cells with immune checkpoint blockades.

## Introduction

Lung cancer is a major cause of cancer-related deaths and the second most diagnosed cancer globally (1). Non-small cell lung cancer (NSCLC) accounts for 80 to 90% of all lung cancer diagnoses in the United States (2), and in Europe (3), with lung adenocarcinoma (LUAD) and lung squamous cell carcinoma (LUSC) being the two most prevalent NSCLC subtypes (4). Numerous variables play a role in the development of NSCLC. Smoking and secondhand smoke exposure are key risk factors (5). Despite remaining uncertain, marijuana and e-cigarette usage have been related to a possible risk of NSCLC (6). Additionally, environmental and professional exposure to asbestos, radon gas, air pollution, and certain pollutants are strong risk factors associated with NSCLC development (7), in addition to genetic predisposition (8).

Although surgery and chemotherapy (including neoadjuvant and/or adjuvant therapy), as well as radiotherapy, have shown improvements in prolonging overall survival (OS) in NSCLC patients, they still present with severe toxic side effects (9). In the past 20 years, targeted therapies have become the gold standard to treat NSCLC patients with actionable oncogenic alterations, including driver mutations and fusions/rearrangements (10). These patients represent only 15–20% of all NSCLC patients and, while targeted therapies are initially effective, resulting in prolonged progression-free survival (PFS) and improved OS, their efficacy is limited by the emergence of resistance mechanisms (11).

Therefore, developing new treatment approaches that impact on PFS and OS is now the primary focus in NSCLC research. In this context, targeting specific components of the tumor microenvironment (TME) in NSCLC is seen today as the Holy Grail to boost the effectiveness of established drugs and design novel anticancer agents. Drugs targeting cancer associated fibroblasts, extracellular components, immune cells, endothelial cells and the surrounding tumor vasculature have been recently approved by regulatory agencies (12, 13). A dominant trait of NSCLC is the elevated presence of tumor-specific (TSAs) and tumor-associated antigens (TAAs) on the surface of malignant cells (14). Cancer immunotherapy has the goal to overcome TME by eliciting (or re-igniting) an adaptive immune response, especially the T-cell-mediated TSA- and TAA-directed cytotoxicity against cancer cells (15). Immunotherapies include therapeutic vaccines, autologous cellular therapies, and different types of immune modulators, including checkpoint inhibitors, cytokines, T-cell agonists and adjuvants (16). In patients without a driver mutation, immunotherapy in the form of immune checkpoint inhibitors (ICIs) is currently an integral part of NSCLC treatment (17). Programmed cell death ligand-1 (PD-L1) expression is the main predictive biomarker of response to ICIs directed to PD-L1 and to its binding partner, the programmed cell death protein 1 (PD-1) (18). Normally, the five-year OS rate exceeds 25% for patients having advanced NSCLC with a PD-L1 score of 50% or higher (19, 20). This statistics is still superior to the average OS of patients treated with conventional drugs, which rarely exceed 15% (21, 22). Nevertheless, a more reliable marker of response is missing and highly wanted to improve patient stratification (23) considering that a significant fraction of patients fails to benefit from those treatments. Among investigated biomarkers, the tumor mutational burden (TMB) has recently emerged as a major predictor of immunotherapy efficacy in NSCLC (17). However, to elicit effective immune response novel immunotherapeutic approaches are needed in the so-called “cold tumors”, which are generally characterized by a decreased TMB, defective HLA class I antigen processing machinery (APM), reduced T-cell presence, and low PD-L1 expression. Two of the most advanced approaches involve adoptive cell transfer (ACT) with autologous tumor-infiltrating lymphocytes (TILs) and chimeric antigen receptor (CAR)-based therapies. In this scenario, there has been a rise in the number of clinical trials exploring the safety and efficacy of different antigen-based ACTs.

## Targeted therapies

The discovery of targetable genetic alterations has radically changed the approach to NSCLC treatment. With the identification of specific cancer driver mutations, today clinicians can provide individualized therapies that lead to extremely efficient responses in some patients’ subsets when treated for example with the matched targeted tyrosine-kinase inhibitor (TKI) (24, 25).

Targeting of the epidermal growth factor receptor (EGFR) is a paradigm of this type of therapy. Mutations in the *EGFR* gene are prevalent in approximately ~15% of patients with NSCLC (~50% in Asians), and they play a primary role in disrupting cellular functions such as cell growth, survival, invasion, and angiogenesis. Mutations in EGFR can lead to continuous activation and independence from ligands, particularly in the ATP-binding region of the tyrosine kinase domain. Notably, common EGFR mutations, including exon 19 deletions and the L858R mutation on exon 21, have been associated with heightened sensitivity to EGFR TKIs (24, 26–29). First-generation anti-EGFR TKIs, like gefitinib (NCT01203917) (30) and erlotinib (NCT00446225) (31), have demonstrated superior response rates and longer PFS (9.2–13.1 months) than traditional cytotoxic therapies in treatment-naive patients with EGFR mutations (24, 31–37). Second-generation inhibitors, such as afatinib (NCT00949650; NCT01121393) (38) and dacomitinib (NCT01774721) (39), target EGFR but also the receptor tyrosine-protein kinase erbB-2 (HER2) and erb-4 (HER4), resulting in improved progression-free survival compared to gefitinib (24, 38–40). The most common acquired resistance to first-generation TKIs arises due to the additional EGFR mutation T790M in exon 20 (24, 41, 42); other resistance mechanisms to targeted therapy include *HER2* amplification, mutations in the MET proto-oncogene receptor tyrosine kinase (*MET*), the B-Raf proto-oncogene serine/threonine kinase (*BRAF*), the ROS proto-oncogene 1 receptor tyrosine kinase (*ROS1*), and the phosphatidylinositol-4,5-bisphosphate 3-kinase catalytic subunit alpha (*PIK3Cclinical A*) genes (24, 43). Interestingly, resistance to TKIs can also cause lung adenocarcinoma to evolve into small-cell lung cancer (SCLC): roughly, 3% to 10% of EGFR-mutated NSCLC could shift to SCLC clinical subtype (44). Third-generation EGFR inhibitors selectively target the original activating mutations, in addition to the T790M resistance mutation. Osimertinib, a representative third-generation anti-EGFR TKI, while exhibiting higher response rates and longer PFS than platinum-based chemotherapeutic treatment (45), has also shown efficacy in NSCLC patients with EGFR-T790M mutations after progression to treatment with first-generation TKIs (24, 46, 47). FLAURA clinical trial (NCT02296125) comparing osimertinib to erlotinib or gefitinib as a first-line treatment in advanced NSCLC patients with EGFR mutations revealed that osimertinib significantly improved PFS, establishing it as the preferential first-line treatment option (24, 48, 49). Acquired resistance to third-generation anti-EGFR TKIs can also occur through the C797S mutation (24, 50). Triple mutants carrying the original sensitizing mutation, plus acquired T790M and C797S mutations, show strong resistance to TKIs from all three generations (24, 51). Promising approaches for these triple mutants include allosteric inhibitors like EAI045 in tumors with the L858R-sensitizing mutation and the anaplastic lymphoma kinase (ALK) inhibitor brigatinib in tumors with exon 19 deletion. These inhibitors can be combined with cetuximab, an anti-EGFR monoclonal antibody, to target tumors harboring the triple mutant (24, 52, 53).

ALK-positive tumors represent about 4% of lung cancers, and generally appear in adenocarcinoma NSCLC of younger non-smoker patients (54). Typically, the *ALK* gene is rearranged with the echinoderm microtubule-associated protein-like 4 (*EML4*) gene, forming the EML4-ALK fusion protein (24, 55). Crizotinib, which is an oral inhibitor that targets mutations in the kinase domain of ALK (NCT00932893) (56), MET, and ROS1 (NCT00585195) (24, 57, 58), also shows effectiveness in treating NSCLCs with *ALK* fusion by improving 62% PFS and response rate, compared to traditional chemotherapy (24, 59–62). Second-generation ALK inhibitors like ceritinib (63), brigatinib (NCT02737501) (64), and alectinib (NCT02075840) (60) have been shown to be effective in the second-line setting after resistance to crizotinib, which is typically used as first-line treatment for ALK-mutated patients (65). As a second-line treatment, alectinib showed an objective response rate (ORR) of 45% and PFS of 8 to 12 months, and brigatinib showed an ORR of 45% to 54% with a PFS of 9.2 to 12.9 months (65). Also, clinical studies have shown higher ORR and median PFS for alectinib than crizotinib in previously untreated patients with ALK-positive NSCLC, establishing alectinib as a viable first-line option (59, 65). Resistance to ALK inhibitors can arise from various alterations in the ALK gene, such as mutations and amplification, or through the activation of alternative signaling pathways, like the EGFR and the mitogen-activated protein kinase (MAPK) cascade (66). Among all resistance mechanisms, secondary ALK mutations are the main drivers of resistance to second-generation TKIs (67). The most common ALK resistance mutation observed in patients treated with second-generation TKIs is the G1202R. This mutation confers *in vitro* resistance to all available ALK inhibitors except lorlatinib. Lorlatinib, a potent third-generation ALK inhibitor, is effective against most known ALK resistance mutations, and it has demonstrated efficacy in patients previously treated with up to three lines of ALK inhibitors, providing a potential treatment option for overcoming resistance (NCT01970865) (68, 69).

As mentioned before, crizotinib is an oral inhibitor that targets ALK, MET, and ROS1 tyrosine kinases. *ROS1* gene can undergo rearrangements and typically fuses with the *CD74* gene, which causes the receptor tyrosine kinase domain to become persistently active (70). Ceritinib and lorlatinib (NCT01970865) (69) also exhibit notable efficacy in ROS1-positive tumors (68, 71, 72). Resistance to crizotinib in cases of *ROS1* rearrangements can arise through various mechanisms, including secondary mutations, most notably the G2032R mutation. Additionally, resistance may occur due to the activation of wild-type EGFR signaling or mutations in the Kirsten rat sarcoma viral proto-oncogene (*KRAS*) and the KIT proto-oncogene receptor tyrosine kinase (*KIT*) genes (73, 74).

Additional targetable gene alterations in NSCLC include BRAF and HER2 mutations, as well as rearrangements in the RET proto-oncogene (*RET*), and fusions involving the neurotrophic receptor tyrosine kinase (*NTRK*) genes 1-3 (*NTRK1*, *NTRK2*, *NTRK3*) (24). Roughly half of the NSCLC patients with a BRAF mutation carry the V600E activating mutation in exon 15 (75, 76). This mutation indicates sensitivity to BRAF inhibitors like vemurafenib (NCT01524978) (77) and dabrafenib, alone or combined with trametinib (NCT01336634) (78–82). Acquired resistance mechanisms to BRAF inhibitors typically include secondary BRAF alterations (e.g., splice variants), activating mutations in the mitogen-activated extracellular signal-regulated kinase (MEK) and the proto oncogene serine/threonine protein kinase CRAF, neurofibromatosis type 1 (NF1) gene loss, and sustained activation of the MAPK pathway thought bypassing signaling via other tyrosine receptor kinases like EGFR or MET (83, 84). Targeted therapies against HER2 and RET alterations have shown moderate activity compared to other targeted treatments, likely due to their dominant role as drivers of tumor growth (85–89). **Table 1** summarizes the major TKIs, their targets, and associated resistance mechanisms.

**Table 1:** Summary of major tyrosine kinase inhibitors (TKIs) and related therapeutic targets in non-small cell lung cancer, along with most frequent mechanisms of acquired resistance.

TKI	Molecular target	Associated resistance mechanisms
Gefitinib	EGFR	• Mutation T790M in exon 20 of the *EGFR* gene • *HER2* amplification • Mutations in the *MET* gene • Mutations in the *BRAF* gene • Mutations in the *ROS1* gene • Mutations in the *PIK3C* gene
Erlotinib		
Afatinib		
Dacotinib	EGFR, HER2, HER4	
Osimertinib	wild-type EGFR and EGFR-T790M mutation	• Acquisition of the C797S mutation
Crizotinib	AKL	• Alterations in the *ALK* gene: mutations and amplification • Activation of alternative signaling pathways: EGFR and the MAPK cascade
Ceritinib		• Secondary ALK mutations: the most common is the G1202R
Brigatinib		
Alectinib		
Lorlatinib		
Crizotinib	ROS1	• Secondary mutations: most notably the G2032R mutation • Activation of wild-type EGFR signaling • Mutations in the *KRAS* gene • Mutations in the *KIT* gene
Ceritinib		
Lorlatinib		
Vemurafenib	BRAF	• Secondary BRAF mutations • Activating mutations in the *MEK* gene • Activating mutations in the *CRAF* gene • *NF1* gene loss • Sustained activation of MAPK cascade: signaling bypass via EGFR and MET
Dabrafenib		
Trametinib and Dabrafenib		

ALK: Anaplastic Lymphoma Kinase; BRAF: B-Raf Proto-Oncogene Serine/Threonine Kinase; CRAF: Raf Proto-Oncogene Serine/Threonine-Protein Kinase; EGFR: Epidermal Growth Factor Receptor; HER2: Human Epidermal Growth Factor Receptor 2; KIT: KIT Proto-Oncogene Receptor Tyrosine Kinase; KRAS: Kirsten Rat Sarcoma Viral Proto-Oncogene; MAPK: Mitogen-Activated Protein Kinase; MEK: MAPK/ERK Kinase; MET: MET Proto-Oncogene, Receptor Tyrosine Kinase; NF1: Neurofibromatosis type 1 1; PIK3CA: Phosphatidylinositol-4,5-Bisphosphate 3-Kinase Catalytic Subunit Alpha; ROS1: ROS Proto-Oncogene 1, Receptor Tyrosine Kinase.

## Tumor microenvironment and immunotherapy

### Immune suppression within the TME

The genetic alterations that initiate and drive tumor growth not only affect the behavior of cancer cells but also shape the TME composition, by affecting both immune cells function and non-cellular components of the extracellular matrix (90).

LUAD tumors exemplify the intricate signaling networks employed by cancer cells to coerce non-malignant cells for their advantage. These types of lung cancer which are characterized by a high burden of clonal neoantigens that promote an “inflamed” TME, with an abundance of activated effector T cells, increased expression of proteins, such as the chemokine (C–X–C motif) ligand-9 (CXCL-9) and -10 (CXCL-10), involved in antigen presentation and T-cell migration (91). At the same time, an inflamed TME also expresses negative regulators of T-cell activity, like the lymphocyte-activation gene 3 (LAG-3), PD-L1, PD-1 and T cell immunoglobulin and ITIM (TIGIT). The bright side is that LUAD tumors with a high TMB and elevated PD-L1/PD-1 expression may respond well to ICI treatment (91). Also, an important cellular alteration associated with a high TMB is the loss of mismatch repair function followed by an increased microsatellite instability, the last being associated with improved responses to ICIs (92). Although tumors with microsatellite instability had shown some promising responses to ICIs (93), recent findings revealed that genome instability can fuel resistant phenotypes of tumor cells to both targeted therapy and ICI (94). Nevertheless, genetic alterations can negatively affect the TME in other ways. For instance, the inactivation of the tumor suppressor gene serine/threonine kinase 11 (*STK11*) in KRAS-mutated LUAD shifts the TME toward tumor infiltration by immunosuppressive neutrophils and the reduction of PD-L1 expression in cancer cells and less TILs (95).

Research on TILs has provided valuable understanding about the role of lymphocytes within the tumor stroma and their contribution to the development of an immunogenic response (96–98). In fact, a high density of T lymphocytes within the tumor bulk, including CD4+ and CD8+ cells, typically correlates with improved outcomes (97, 99). Specifically, CD8+ T cells and M1 macrophages have been associated with favorable prognosis and prolonged OS (97). A recent study focused on the association between the presence of CD8+/PD-L1+ TILs and the TMB, indicating an immunosuppressive TME, in those patients that were more likely to respond to anti-PD-1 therapy (100). To investigate more deeply the underlying mechanisms, Caushi et al. utilized single-cell transcriptomics to analyze specific TILs targeting mutation-associated neoantigens (MANAs) in NSCLC tumors from patients enrolled in a clinical trial with nivolumab alone or in combination with ipilimumab (101–103). The study revealed that MANA-specific CD8+ T cells were more abundant within the TME compared to the normal lung tissue of the same patient. Moreover, MANA-specific T cells from responsive patients exhibited an increased expression of genes associated with T cell memory, including the interleukin 7 receptor (*IL7R*), T-cell factor/lymphoid enhancer-binding factor 7 (*TCF7*), and the granzyme K (*GZMK*). Conversely, MANA-specific T cells from non-responsive patients predominantly expressed genes linked to T cell dysfunction, such as TOX high mobility group box family member 2 (*TOX2*), cytotoxic T-lymphocyte antigen 4 (*CTLA4*), hepatitis A virus cellular receptor 2 (*HAVCR2*), and the ectonucleoside triphosphate diphosphohydrolase 1 (*ENTPD1*) gene (102).

While some patients that are responsive to immunotherapy exhibit a tumor with a TME characterized by the presence of TILs, macrophages, and dendritic cells (DCs), other patients have tumors with a so-called “cold” TME, which is less permeated by TILs, or show an “altered” TME, where TILs are primarily found at the tumor’s edge (104). Comprehensive analyses integrating spatial histology and genetic information have shown that tumors with multiple immune cold regions are characterized by a higher risk of relapse (105). Furthermore, low TIL counts were found associated with reduced efficacy of ICI treatment and resistance to immunotherapy (105). These findings emphasize the importance of understanding the composition of the TME and spatial distribution of its components in predicting therapy response and patients’ outcome. Dysfunctional CD8+ TILs, referred to as “burned-out” (Ebo) TILs, have been identified in advanced NSCLC patients (106). Ebo TILs showed heightened proliferation and activation markers but reduced production of interferon-gamma (IFNγ). Notably, Ebo TILs expressing elevated PD-1, T-cell immunoglobulin, mucin-domain containing-3 (TIM-3), and LAG-3 were associated with resistance to anti-PD therapy in NSCLC patients (106). Furthermore, the presence of inhibitory receptors on TILs, including PD-1, TIM-3, the cytotoxic T-lymphocyte antigen 4 (CTLA-4), LAG-3, and B and T Lymphocyte associated (BTLA) receptor, associate to a progressively impaired capacity of T cells to respond to polyclonal activation (107).

### Tertiary lymphoid structures in NSCLC

The so called ‘Tertiary Lymphoid Structures’ (TLS) are organized aggregates of ectopic lymphoid tissues that form within the TME, and consisting of germinal centers where cognate T cells and B cells interact to develop the anti-tumor adaptive immune response. TLS are essential to promote an antigen-specific immune response at sites of chronic inflammation, after consecutive antibody somatic hypermutation and affinity maturation happening (96, 108, 109). Several studies have associated the presence of B cells in TLS with more favorable outcomes in NSCLC (110–114). B cells have the capacity to activate and proliferate into plasma cells, which can generate tumor-specific antibodies that attack tumor cells and trigger the complement system, enhancing both antibody-dependent cytotoxicity (ADCC) and antibody-dependent cellular phagocytosis (ADCP) (115, 116). In a recent study, it was demonstrated that TLS maturation is associated with major pathological response, which can be used as an independent predictor of disease free survival (DFS) in resectable neoadjuvant chemoimmunotherapy-treated NSCLC (117). The study collected formalin-fixed paraffin embedded tissues from patients with resectable NSCLC, divided in three cohorts based on treatment: naïve, neoadjuvant chemoimmunotherapy, and neoadjuvant chemotherapy. Among the three cohorts, neoadjuvant chemoimmunotherapy-treated NSCLCs showed the highest TLS maturation and abundance; both the maturation and abundance of TLS were significantly correlated with major pathological response in both the neoadjuvant chemoimmunotherapy and the chemotherapy group. Patients with high maturation and abundance of TLS exhibited better DFS in all the three cohorts. TLS maturation was also an independent predictor for DFS in the neoadjuvant chemoimmunotherapy and treatment naïve group (117).

### Novel strategies and targets in NSCLC

Natural killer (NK) cells are also very important in regulating the interplay between cancer cells and the TME in NSCLC patients treated with ICIs. NK cells, specifically the non-cytotoxic CD56-bright-CD16-subset, express immunoactivation markers that accumulate in the stroma of NSCLC tumors, including the NK-specific triggering receptor (NKp44), the CD69, and the human leucocyte antigen DR (HLA-DR) protein (118, 119). Importantly, PD-1 is also expressed by NK cells (120). To optimize ICI treatment, a randomized controlled trial in NSCLC patients with positive expression of PD-L1 explored the combination of *in vitro* expanded allogenic NK cells with anti-PD-1 therapy. This novel approach yielded promising results, as it improved OS and PFS, as compared to anti-PD-1 therapy alone (121).

ICI resistance in NSCLC patients associates with an increased number of immunosuppressive cells, including regulatory T cells (Treg), myeloid-derived suppressor cells (MDSC), tumor-associated macrophages (TAM)-M2, and neutrophils (122–124). Treg cells inhibit T cell responses and are associated with poor clinical outcomes in lung cancer patients (125). Studies have shown an increase in PD-1+ Treg cells in patients who do not respond to anti-PD-1/PD-L1 ICI, suggesting that the balance of PD-1 expression between CD8+ T cells and Treg cells in the TME can be used as a more accurate predictor of ICI therapy effectiveness rather than the expression of PD-L1 itself or the TMB (126). Hence, targeting Treg cells could potentially enhance the efficacy of ICI treatment for lung cancer (127, 128). MDSCs can induce immunosuppression through various mechanisms, including the production of molecules that hinder T cell function and interfere with T cell movement (129). MDSCs expressing specific receptors, like CD39 and CD73, have been found in NSCLC tumor tissue, being associated with disease progression (130, 131). Additionally, a 2020 study suggested an association between PD-L1 protein expression on macrophage cells and improved OS in patients treated with anti-PD-1 therapy (132).

Tumor associated neutrophils (TAN) in the TME may also contribute to immune suppression and resistance to ICI treatment in NSCLC, by mediating the suppression of Th1 and cytotoxic T lymphocytes. In particular, in these NSCLC patients, arginase-1 (ARG1)-expressing neutrophils negatively correlates with the proportion of CD8+ T cells, while ARG1-expressing granulocytic cells can lead to CD3ζ chain downregulation on T cells though L-arginine depletion and ultimately inhibit T-cell proliferation and cytokine secretion. In addition to the direct inhibition of effector T cell functions, TANs have also been implicated in regulatory T cell (Treg) recruitment (133–136) to improve T cell activation and ICI therapy response TME is also influenced by the family of vascular endothelial growth factor (VEGF) proteins and their receptors (VEGFRs). VEGF signaling plays a key role in tumor-induced angiogenesis and in promoting tumor growth in NSCLC patients (137). VEGF also influences the immune response within the TME. It can suppress the activity of antigen-presenting cells (APCs), including DCs, NK cells, and T cells. At the same time, VEGF enhances the suppressive effect of Tregs, TAMs, and MDSCs. This combination creates an immunosuppressive microenvironment that allows the tumor to evade the immune system’s surveillance. During the past years, the hypothesis that targeting VEGF could enhance the effectiveness of ICI has also been explored (137). For example, in a phase 3 study (NCT02366143) bevacizumab, an anti-VEGFA antibody, was combined with atezolizumab, an anti-PD-L1 antibody, plus chemotherapy in patients with metastatic lung cancer who had not previously received chemotherapy. This combination significantly improved OS and PFS, irrespective of their PD-L1 expression levels or EGFR or ALK genetic alteration status (138).

Lately, there has been a concerted effort to identify reliable TME-based markers to predict the effectiveness of immunotherapy for lung cancer. One notable tool is the “immunoscore” (IS), which assesses the presence of T lymphocytes within the tumor tissue (139). This digital test examines various T cell subpopulations in both the central and peripheral regions of the tumor, generating a score that ranges from IS 0 (low immune cell density) to IS 4 (high density in both areas). The IS has shown promising results in several cancer types, including NSCLC, where a higher IS score is associated with better survival outcomes (140). CD8+TILs have emerged as a potent biomarker for differentiating patients with more favorable PFS following immunotherapy with ICI (141–144). Expression of CD8 can act either as a prognostic or a predictive factor of clinical outcome: in NSCLC patients not treated by immunotherapy, high CD8A expression is associated with longer OS, while in NSCLC patients treated with anti PD1, high CD8 expression is associated with longer PFS (145). Another approach to predict the effectiveness of immunotherapy for lung cancer involves the generation of specific gene signatures to characterize the TME, aka the “immune gene signatures” (146, 147). These signatures consist of lists of genes that indicate if enriched/depleted (i.e., coherently up- or down-regulated, respectively, in a tumor sample), the presence of specific immune or stromal cell populations or describe TME-cell activation states. High-throughput technologies like microarray and RNA sequencing have facilitated the development of computational algorithms capable of predicting non-cancer cell infiltration in tumors (148–154). These algorithms generate scores that define the grade of immune and stromal cell infiltration, providing valuable insights into cancer molecular and immune characteristics and its potential impact on ICI response. As a matter of fact, the gene signature scores provide specific TME-based markers that offer valuable information on tumor heterogeneity, and enable meaningful comparisons between different tumor samples (155), showing great promise in advancing our understanding of immunotherapy response in lung cancer.

ICIs, such as monoclonal antibodies targeting CTLA-4 and antibodies against PD-1 or PD-L1, have opened new avenues for managing lung cancer (156–159). So far, the Food and Drug Administration (FDA) has approved the following ICIs: the anti-PD-1 antibodies nivolumab (NCT01642004) (160), pembrolizumab (NCT01295827) (19), and cemiplimab (NCT03409614) (161), as well as the anti-PD-L1 antibodies atezolizumab (NCT01903993) (162), durvalumab (NCT02125461) (163), avelumab (NCT02576574) (164), and sugemalimab (NCT03789604) (165, 166). Also, FDA has approved ipilimumab, an anti CTLA-4 antibody (NCT02477826) (167).

Immunotherapy has revolutionized the treatment approach for advanced NSCLC, due to the favorable safety profile of most ICIs and improved survival outcomes, which make these drugs particularly effective for patients who experience disease progression after initial cytotoxic therapy (160, 168), and a promising first-line treatment option (169).

In the first-line setting, pembrolizumab has become the standard of care for metastatic NSCLC patients with tumor expression of PD-L1 over 50%, a condition that may occur in approximately 30% of NSCLC cases (170). Pembrolizumab has significantly improved ORR, PFS, and OS compared to platinum-based cytotoxic therapy (171, 172). On the other hand, nivolumab did not show similar benefits in PFS or OS among patients with PD-L1 expression levels above 5% (171). Notably, in patients treated with nivolumab who had both high PD-L1 expression and high TMB, the objective response rate was 75%, suggesting that a predictive value for both TMB and PD-L1 expression in determining the efficacy of ICI therapy exists (169).

Even further, combination of ICI with cytotoxic chemotherapy has shown enhanced treatment outcomes in NSCLC (173). Combination of pembrolizumab with carboplatin and pemetrexed has demonstrated improved ORR and PFS compared to cytotoxic therapy alone, making it a promising first-line treatment option for advanced NSCLC patients (174). A detailed meta-analysis, including all randomized controlled trials published before February 2022, showed that the combination of ICIs with chemotherapy is much more effective in enhancing PFS, ORR, and OS in NSCLC patients (175).

Several novel immune checkpoints with promising therapeutic potential have recently been identified, among them the LAG-3, T-cell immunoglobulin and TIM-3, B7 Homolog 3 (B7-H3), and T cell immunoglobulin and TIGIT domain (176). To target LAG-3, researchers are exploring the use of a soluble dimeric recombinant LAG-3 (eftilagimod alpha, or IMP321), which stimulates DCs through the binding with the major histocompatibility complex (MHC) class II receptor, leading to sustained immune responses when combined with anti-PD-1 therapy in patients with previously untreated unresectable or metastatic NSCLC [NCT03625323 (177)]. Another approach involves bispecific antibodies (BsAbs) simultaneously targeting LAG-3 and PD-1 [NCT04140500 (178); NCT03219268 (179)]. Besides, the anti-LAG-3 antibody relatlimab (BMS-986016) has shown promising results in the phase III trial RELATIVITY-047 (NCT03470922), where treatment-naïve patients with metastatic melanoma who received nivolumab plus relatlimab demonstrated significantly longer median PFS than those who received nivolumab plus placebo (180). Considering these encouraging findings, further investigations are underway to evaluate the dual blockade in other solid tumors, including NSCLC (NCT04623775 (181).

TIM-3, known for its presence on CTL, NK, Treg, DC, and macrophages (where it promotes M2 polarization), is a critical immune checkpoint being investigated in various clinical trials for solid tumors, including NSCLC (182). Monoclonal antibodies targeting TIM-3 alone or combined with anti-PD-1 are being studied in trials such as the studies NCT03652077 (183) and NCT02608268 (184). Moreover, bispecific antibodies (BsAbs) that can simultaneously block TIM-3 and PD-1 are also explored in ongoing trials involving NSCLC patients [NCT03708328 (182, 185); NCT04931654 (186)].

The B7 homologous 3 (B7-H3), also known as CD276, is a transmembrane protein commonly expressed by cancer cells that act as an immune checkpoint, allowing cancer cells to evade immune surveillance. Researchers believe that B7-H3 expression might play a role in the resistance to anti-PD-1/PD-L1 therapy in NSCLC (187, 188). There are currently three ongoing clinical trials evaluating the use of an anti-B7-H3 antibody in combination with either anti-PD-1 or anti-CTLA-4 for advanced solid tumors, including NSCLC (NCT03729596; NCT02475213; NCT02381314) (189–191). As detailed below, novel immunotherapies targeting B7-H3 are considered very promising in enhancing the clinical response and overcoming resistance in NSCLC patients, including the use of chimeric antigen receptor (CAR)-T cells (192).

The immune receptor TIGIT is expressed by several immune cells, including CD8+ T cells, CD4+ T cells, and NK cells, and it is a candidate target of vibostolimab antibody in NSCLC (12, 193). The phase I study NCT02964013 (194) was the first to investigate safety and efficacy of vibostolimab as a monotherapy or in combination with pembrolizumab for treating advanced solid tumors, including NSCLC (12, 194). Another study, the phase II CITYSCAPE trial (NCT03563716), combined another TIGIT inhibitor, tiragolumab, with the anti-PD-L1 atezolizumab (195). The study showed that tiragolumab plus atezolizumab is a promising immunotherapy combination for the treatment of NSCLC. In particular, tiragolumab plus atezolizumab showed a considerable improvement in ORR and PFS compared with placebo plus atezolizumab in patients with chemotherapy-naive, PD-L1-positive, recurrent and metastatic NSCLC. Tiragolumab plus atezolizumab was well tolerated, with a safety profile generally similar to that of atezolizumab alone (195).

It is important to note that the safety profile of these ICIs in combination may differ from when ICIs are coupled to chemotherapy, particularly regarding immune-related adverse events. Plus, despite initial encouraging results in certain patients, many lung tumors exhibit intrinsic resistance to immunotherapies. Hence, the next challenge will lie in identifying rational combinations that can enhance treatment responses and delay the onset of resistance (196).

## Latest immunotherapy-based strategies for NSCLC

To elicit an effective immune response in so-called ‘immune desert tumors’, which are devoid of lymphocyte infiltration, little TMB, and low PD-L1 expression, the main challenge is to attract effector T cells to the TME and present them with the tumor antigens. Among those novel approaches, the two most promising include the adoptive cell transfer (ACT), based on autologous T cells derived from TILs, and CAR-T therapies (197). However, identifying NSCLC-specific or unique cell surface antigens is necessary for the exploitation of CAR-T approach. TAAs that are frequently found to be overexpressed in NSCLC include the Mucin 1 (MUC-1), the carcinoembryonic antigen (CEA), the New York esophageal squamous cell carcinoma 1 (NY-ESO), and the melanoma-associated antigen 3 (MAGE-A3) (198–200). The problem is that these antigens are also commonly expressed in normal lung cells. As a consequence, these TAAs are potentially not very much immunogenic and can be also used by the tumor to develop immune tolerance, which results in reduced responsiveness to ICIs (201).

On the other hand, TSAs are unique to cancer cells and result from nonsynonymous somatic mutations. These TSAs represent ideal targets for cellular immunotherapy (202, 203). NSCLC, like other tumors with high TMB, have been shown to possess a significant number of TSAs arising from various somatic mutations, including driver genes like tumor protein 53 (TP53), Kristen Rat sarcoma viral oncogene homolog (KRAS), cyclin-dependent kinase inhibitor 2A (CDKN2A), AT-Rich Interaction Domain 1A (ARID1A), Neurogenic locus notch homolog protein 1 (NOTCH1), myelocytomatosis oncogene (MYC), SWI/SNF related, matrix associated, actin dependent regulator of chromatin, subfamily A, member 4 (SMARCA4), and retinoblastoma 1 (RB1) (204, 205). TILs can recognize these neoantigens, and their density has been linked to a more favorable prognosis, higher cytotoxic T lymphocyte (CTL) content, and increased benefit from ICI (206, 207). Despite the inherent challenges, recent technological advancements, such as the MANA Expansion of Specific T cells platform, has already been used to detect and monitor peripheral and intratumoral MANA-specific T cell responses in NSCLC patients with acquired resistance to checkpoint blockade (208). Notably, CTLs that are specifically directed against peptides derived from oncogenic driver mutations, such as TP53 R248L and BRAF N581I (208, 209), have been detected, offering potential avenues for new targeted immunotherapies in lung cancer.

Cancer vaccines may augment the body’s T cell and B cell response against TAA or TSA. There are different mechanisms to stimulate the immune system and generate an effective anti-tumor response (210). One common approach involves the use of DCs collected from a patient’s blood, loaded with TSAs derived from the tumor, and then administered back to the patient. These DCs migrate to lymphoid organs, where they interact with host immune cells, including T- and B- cells, leading to their activation and enhancing of the immune response against tumor cells (211). A different strategy entails using whole-cell preparations obtained from cancer cells that have undergone either inactivation or genetic modification. The inactivated cancer cells are recognized by the host immune system, triggering an immediate nonspecific inflammatory response (212). Induced pluripotent stem cells (iPSCs) derived from primary fibroblasts exhibit genetic and transcriptomic similarities with cancer tissues, encompassing numerous cancer-associated genes as well as over 100 TAAs and TSAs, which are protein markers detectable by the immune system (213, 214). Recently, research in mice has explored the use of iPSCs as a source of tumor- and patient-specific antigens to direct the immune system to target cancer (213). Kooreman further developed the idea of an iPSC-based anticancer vaccine by combining irradiated autologous mice iPSCs with the Toll-like receptor 9 (TLR9) agonist, CpG oligodeoxynucleotide. Using autologous iPSCs helped reduce immune reactions caused by MHC mismatches, while the addition of the CpG adjuvant enhanced the activation of antigen-presenting cells like dendritic cells (213, 215). In a humanized mouse model of lung cancer, vaccination with iPSCs and CpG led to an increase in splenic APCs, cytotoxic T cells, circulating effector/memory CD4+ and CD8+ T cells, and tumor-infiltrating CD8+ T cells, while reducing regulatory T cells (Tregs). This immune response contributed to effective tumor growth suppression, with tumor antigen-specific T cells playing a pivotal role. This was demonstrated by the protective effect seen after transferring spleen T cells from vaccinated mice to unvaccinated ones. The immunity triggered by iPSCs was thought to stem from shared gene expression patterns between iPSCs and lung adenocarcinoma stem cells (213, 216). With an alternative approach, viral or bacterial -based cancer vaccines are used to directly activate the immune response against TSAs and TAAs: the antigens are either expressed by a virus or bacteria, delivered to infect the host cells, which process the antigen and present it to activate the T cell response (217).

Numerous clinical trials are currently investigating different vaccines targeting specific NSCLC antigens like MAGE-A3, CEA, mesothelin, KRAS proto-oncogene (KRAS), New York esophageal squamous cell carcinoma 1 (NY-ESO-1), and telomerase (TERT), as well as immunomodulatory enzymes such as indoleamine 2,3-dioxygenase (IDO) and arginase-1, in lung cancer patients (199, 218). Some of these cancer vaccines are combined with ICIs in phase I/II studies (NCT04908111, NCT02879760, NCT03562871, NCT05202561, NCT04117087, NCT01935154; NCT03689192, NCT03970746, NCT02187848) (219–227).

Importantly, the success of COVID-19 messenger RNA (mRNA) vaccines has shown how effective this type of vaccine can be, how fast it can reach a clinical stage and the ease of production at global scale (228). This approach involves the use of synthetic mRNA sequences, either alone or in combination with other molecules, which encode proteins found in cancer cells. The expression of these proteins triggers immune reactions targeting tumor antigens, such as the production of antibodies and cytotoxic T cells (229). Early studies, like the NCT00004604 trial conducted at the beginning of 2000s to evaluate mostly safety and dose-limiting toxicities of mRNA-based vaccines using DCs, proved quite ineffective in terms of clinical response and disease progression (230). At present, an upgraded form of these vaccines is being introduced, showing improved efficacy and tolerability. For example, the mRNA-based vaccine CV9201, tested in the NCT00923312 phase I/IIa trial, showed promising results for advanced NSCLC patients (231). Also, the CV9202 mRNA-based vaccine was used in combination with radiation therapy in stage-IV NSCLC patients in the NCT01915524 study showing increased antigen-specific immune responses, and stable disease achieved in 46.2% of patients (232). On balance, while mRNA vaccines offer substantial benefits over conventional options thanks to their high efficacy, minimized toxicity, accelerated manufacturing, and reliable administration, there are some potential limitations that could overshadow their widespread use (233). For example, these constraints include lack of stability and reduced translation rates upon delivery into target cells, mostly due to inadequate methylation of the mRNA, or small impurities in the preparation process (234). Intrinsic immunogenicity of the construct can also impair the stability of the mRNA vaccine, and ultimately cause decreased translation. Increasing the capping efficiency and preventing de-capping through the incorporation of modified nucleosides or pseudouridine can actually avoid recognition by the innate immune system and prevent mRNA destruction (233). Inefficiency of *in vivo* delivery in target tissues, including the lungs, is another major issue that limits the potential of mRNA vaccines and requires further optimization of the vectors (viral, non-viral, cell-based, lipid vesicles). A robust targeting of mRNA has been achieved in pulmonary tissues, for example, by the use of nanoparticles (235). Furthermore, the specificity of the mRNA-encoded polypeptide, although tailored to generate only the specific antigen of interest, may not only limit the efficacy of inducing a robust immune response in the cancer tissue but can also result in serious side effects, typically including uncontrolled inflammation and anaphylaxis (236).

The YL202/BNT326 is a novel antibody-drug conjugate ADC designed by BioNTech to target HER3, combining an anti-HER3 monoclonal antibody with a topoisomerase I inhibitor, YL0010014, linked via a tripeptide linker. The results of the phase I trial conducted on advanced or metastatic non-small cell lung cancer (NSCLC) patients bearing EGFR-activating mutations, as well as HR-positive, HER2-negative breast cancer (BC) that had previously received third-generation tyrosine kinase inhibitors (TKIs) or CDK4/6 inhibitors and at least one line of chemotherapy were recently presented at the American Society of Clinical Oncology (ASCO) 2024 Annual Meeting held in Chicago. Six different doses of YL202/BNT326 were tested, using a dose-escalation approach, followed by additional dosing in selected cohorts. Safety and tolerability were the primary endpoints, with secondary endpoints including pharmacokinetics and efficacy. In terms of efficacy, 46 patients were evaluable for tumor response. At doses levels 3 to 5, the overall response rate (ORR) was 41.0%, and the disease control rate (DCR) was 94.9%. In breast cancer patients, the ORR reached 54.5%, with an impressive DCR of 100% (237).

## ATMPs for lung cancer: from ACTs to CAR-Ts

Another version of antigen-specific immunotherapy is the adoptive T cell transfer (ACT) of lymphocytes that exhibit antitumor activity. The concept of ACT involves *ex vivo* activation of the patient’s own immune cells, before transferring them back to the patient to recognize and eliminate cancer cells (197). ACT therapies encompass different methods, including adoptive transfer of TILs or genetically engineered T cells with retargeted specificity, such as affinity-enhanced αβ-T-cell-receptor (TCR) and CAR (197). Compared to vaccine-based strategies, ACT provides patients with pre-activated effector cells, eliminating the need for T cell priming in patients who have compromised immune systems or have developed immune tolerance to tumor antigens (238). In current strategies for targeting advanced NSCLC, ACT is employed with engineered T cells directed against specific TAA, like NY-ESO-1/LAGE-1, often in combination with ICI (NCT03709706) (239). ACT approaches using TCR show some challenges, however, since they may be susceptible to tumor escape due to immunoediting processes, through which tumor clones develop mechanisms to evade antigen presentation, such as loss of antigenicity and/or loss of immunogenicity. Loss of antigenicity can occur either through the acquisition of defects in the antigen processing and presentation, or through the loss of immunogenic tumor antigens: both mechanisms lead to a lack of immunogenic peptides presented in the context of a peptide/MHC complex (240). Malignant cells also can earn additional immunosuppressive properties, such as expression of PD-L1 or secretion of suppressive cytokines (240). To address this issue in particular, CAR-T cells have been developed as an alternative technology to redirect T cell specificity by recognizing intact cell surface proteins, and so bypassing MHC-mediated antigen presentation (241). CARs are engineered receptors formed by three parts: an extracellular antigen recognition domain, usually a single-chain fragment variant (scFv), a transmembrane domain, and an intracellular T cell activation domain (242). All surface-expressed target molecules represent a potential CAR-triggering epitope, so that the genetic modification of T cells with CARs combines the specificity of antibody-like recognition with the cytotoxic activation of T cells (243).

First, construction of a CAR relies on the identification of a suitable antibody that can effectively target a cell surface molecule of interest (244). Carbohydrates and glycolipid antigens on cancer cells are also suitable targets, since they can be recognized by CARs engineered with the antigen-recognition domain derived from monoclonal antibodies (245). The simplest level of CAR structure contains an extracellular domain, connected through a hinge to the transmembrane domain, and an intracellular signaling domain (246). The CAR ectodomain is designed to specifically recognize an antigen on the cancer-cell membrane. Upon engagement, the receptor triggers downstream signaling, resulting in CAR-T cell activation. This leads to a complex network of events comprising transcription factor expression, cell proliferation, survival, and cytokine release which culminates in the execution of a cytotoxic program against the target cell (246).

A hinge sequence is used to connect the ectodomain to the transmembrane (TM) region of the CAR. The hinge region’s length is a crucial parameter in CAR design, since it can modify the flexibility of the scFv and its ability to interact with hidden or distant epitopes on the antigen. On the other end, the specific makeup of the hinge-scFv moiety can be detrimental to CAR efficacy by inducing an unwanted tonic signal of the CAR triggered also in the absence of the antigen, resulting in T cell exhaustion (246). The transmembrane domain serves as an anchor of the CAR to the T cell membrane. Although this domain can also be relevant for CAR-T cell function influencing CAR expression level, stability, signaling, and dimerization with endogenous signaling molecules (247–250). Most transmembrane domains are derived from natural proteins including CD3ζ, CD4, CD8α, or CD28. Endodomain, which transmits the binding signal from the tumor antigen into the T cell, has received most of its attention over the years, resulting in multiple CAR generations. In fact, since the initial development of CARs in 1989, CAR-T constructs can be divided into five generations according to the structure of the endodomain (250, 251). The first-generation CAR design is formed only by Fcγ (the γ-chain from FcϵRI) or CD3ζ (ζ- ζ-chain of the TcR complex) intracellular domain. The durability and persistence of these first generation CARs was not robust because they produced limited amounts of interleukin-2 (IL-2), rendering those CAR T cell dependent on exogenous administration of IL-2 (251). Hence, costimulatory domains were added to the CAR constructs to create the second-generation CARs, which achieved to fully activate T cell proliferation and induce persistent cytotoxicity. Specifically, the insertion in the endodomain of CD28 or 4-1BB was enough to block apoptotic signaling via adequate IL-2 synthesis and complete stimulation of T cells (252). The costimulatory domains CD28 and 4-1BB differ in their functional and metabolic profiles. CARs with CD28 domains differentiate into effector memory T cells and primarily use aerobic glycolysis, while CARs possessing the 4-1BB domain differentiate into central memory T cells and display increased mitochondrial biogenesis and oxidative metabolism (250, 252–254). Thanks to these coreceptors, which yield enhanced persistence, decreased differentiation and exhaustion, prolific expansion, increased cytotoxicity, second generation of CAR constructs have proven to be more effective, compared to first generation CARs, which only included the CD3ζ sequence (252, 253). To further increase the cytokine production and cytotoxicity against tumor cells, a third generation of CARs was designed by adding an extra intracellular signaling sequence in the costimulatory domain such as CD134 or CD137 (251, 252). However, different studies led to ambiguous performance results of third-generation CAR-T use, indicating that clinical outcomes were not always improved compared to the second generation. Moreover, third-generation CAR-T cells have been reported to worsen T cell exhaustion or activation-induced cell death (AICD) (255, 256). A fourth-generation CARs followed based on the second-generation constructs but with the addition of an ILs expression cassette, resulting in the so-called T cells redirected for universal cytokine-mediated killing (TRUCKs), which exhibited remarkable efficacy against different solid tumor types (257). Specifically, IL-12 enhances the response of innate and adaptive immune cells, IFN-γ secretion and the expression of granzyme B and perforin by T cells and NK cells, and suppresses tumor-induced T-regulatory (T-reg) cell proliferation (258, 259). Finally, a fifth-generation CAR-T cells were created to avoid host immune rejection or graft-vs.-host disease against transplanted CAR-T cells (260, 261). These advanced CARs are based on the second-generation of constructs, but they contain a truncated cytoplasmic IL-2 receptor β-chain domain with a binding site for the transcription factor STAT3: the antigen-specific activation of this receptor simultaneously triggers TCR (through the CD3ζ domains), costimulatory (CD28 domain), and cytokine (JAK– STAT3/5) signaling (262).

So far, various NSCLC-associated antigens have been selected as potential candidates for CAR-T cell therapy (263). For instance, CAR-T cells directed against CEA and MUC1, two TSA that are highly expressed in lung cancer, are being evaluated for safety and efficacy in the NCT02349724 (264), NCT04348643 (265), NCT03525782 (266), NCT02587689 (267), NCT05239143 (268) clinical trials (9, 269, 270). Alternatively, the infiltration of CAR-T cells into the tumor is being improved by expressing the C-X-C chemokine receptor type 5 (CXCR5). Also known as CD185, it is the only known receptor for the chemokine ligand 13 (CXCL13), which is abundant in the TME of many NSCLC tumors (271). CAR-T cells engineered to express CXCR5 are now being studied in the trial NCT05060796 (272) for enhanced lymphocytes’ infiltration in the tumor and activation against CXCL13-expressing tumor cell (9, 273–275). Overexpression of EGFR, resulting from EGFR gene amplification and/or mutations, has been detected in a variety of human cancers, including over 60% of NSCLC. This overexpression is linked to tumor recurrence, the formation of new blood vessels, and metastasis (276). The extracellular domain of EGFR present on the surface of tumor cells forms a highly immunogenic and tumor-specific epitope, making it a promising target for CAR-T therapy in NSCLC. Recombinant anti-EGFR CAR-T cells have been developed with specific cytolytic activity against EGFR-positive tumor cells (277). Co-incubation of EGFR-positive tumor cells with anti-EGFR CAR-T cells resulted in the release of high levels of cytokines such as IL-2, IL-4, IL-10, TNF-α, and interferon (IFN)-γ, within 24 hours (276, 278). *In vivo* experiments have shown that these CAR-T cells can proliferate against NSCLC and are found in high proportions among CD8+ cytotoxic T-lymphocyte populations (278). Clinical trials are underway to assess the efficacy and safety of anti-EGFR CAR-T cells in treating advanced NSCLC patients with EGFR-positive tumors (279). For example, a phase I clinical trial at Sun Yat-sen University evaluated anti-EGFR CAR-T cells, modified for the expression of CXCR5, in EGFR-positive patients with advanced NSCLC: among 11 patients being assessed receiving different doses, two showed a partial response, and five remained stable for eight months (280). Another phase I clinical study (NCT03182816) investigated anti EGFR CAR-T cell therapy in NSCLC patients. This trial revealed that EGFR-CAR T cell therapy was feasible and safe in treatment of EGFR-positive advanced relapsed/refractory NSCLC patients, with a progression-free survival of 7.13 months (281).

Mesothelin (MSLN) is another promising CAR target, and assessment of safety and feasibility of anti-MSLN CAR-T cell therapy in the clinical trial NCT02414269 (282, 283) conducted at the Memorial Sloan Kettering Cancer Center is under way. The study focuses on anti-MSLN CAR combined with inducible caspase 9-M28z (iCasp9M28z) suicide system expression and shows promise as a targeted therapy (282). However, the phase I/II trial NCT01583686 led by the US National Cancer Institute (NCI) and assessing anti-MSLN CAR-T cell therapy for patients with MSLN-positive metastatic lung cancer was discontinued due to slow and insufficient patient accrual (284). Nevertheless, there is ongoing interest in the potential of intravenous administration of mRNA-engineered T cells to express anti-MSLN CAR temporarily (285).

Researchers have also developed ROR1-specific CAR-T cells using lentiviral vectors encoding ROR1, scFv/4-1BB/CD3ζ, and truncated EGFR molecules. This engineered CAR-T cell approach effectively eliminated ROR1-positive tumor cells in 3D tumors established from A549 (a non–small cell lung cancer) cell lines on a biological scaffold and an intact basement membrane (286). To evaluate the safety and anti-tumor effects of autologous anti-ROR1 CAR-T cells, a phase I clinical study (NCT02706392) conducted by the Fred Hutchinson Cancer Research Center involved patients with advanced, ROR1-positive, stage IV NSCLC (287). Furthermore, exciting findings from Wallstabe et al. demonstrated the effectiveness of anti-ROR1 CAR-T cells in eliminating both NSCLC and TNBC cells, as shown in organoid tumor models (286) This evidence highlights the potential of anti-ROR1 CAR-T cell therapy as a promising and innovative strategy for treating NSCLC, providing new hope for patients in need of additional treatment options.

Although CAR-T immunotherapy for solid tumors like NSCLC is still in its infancy and has faced limited achievements, it still holds great potential to manage cancers at an advanced stage (288). Importantly, some aspects contribute more than others to hinder CAR-T therapy efficacy in solid tumors:

1. On-target/off-tumor toxicity: CAR-T cells may inadvertently target healthy cells expressing the same antigen as cancer cells, leading to unintended side effects and toxicities (289).
2. Neurological toxicity: some patients may experience neurologic complications due to the activation of CAR-T cells in the central nervous system (289).
3. Cytokine release syndrome (CRS): CAR-T cells can trigger an excessive release of cytokines, causing systemic inflammation and potentially life-threatening complications. CRS is characterized by releasing various inflammatory cytokines upon T cell activation and antigen recognition. This immune response can surge pro-inflammatory molecules like TNF-α, C-reactive protein, IL-2, IL-6, IL-8, and IFN-γ. Consequently, patients may experience fever, fatigue, loss of appetite, hypotension, and, in severe cases, multi-organ dysfunction or even sudden death due to the cytokine storm (289, 290). Early detection and effective management of CRS are vital to safeguard patient well-being during CAR-T cell therapy. Substantial evidence supports using IL-6 pathway inhibitors like tocilizumab or siltuximab as effective treatments for CRS (291, 292). Additionally, infliximab, a TNF-α inhibitor, is a viable option for managing cytokine-related complications (290). By carefully monitoring and promptly intervening, healthcare professionals can minimize the impact of CRS and enhance the overall safety and success of CAR-T cell therapy for patients.
4. The lack of reliable TSA: identifying antigens unique to cancer cells is challenging in solid tumors, reducing the specificity of CAR-T cell therapy.
5. Immunosuppressive TME: solid tumors often create an immune-suppressive environment, hindering the efficacy of CAR-T cells (293). The tumor-associated stroma may form a stumbling block against the entry of T cells, which are already increasingly dimmed by dysregulation of adhesion molecules, aberrant tumor-related vasculature, and mismatching of chemokines and their receptors. Furthermore, TME is characterized by restricted nutrient availability, acidosis, and local hypoxia.
6. Low levels of lymphocytes infiltration within tumor tissue: CAR-T cells may struggle to penetrate and accumulate within the tumor, limiting their ability to target cancer cells effectively.
7. Tumor antigen escape: cancer cells can downregulate or lose the target antigen, evading recognition and destruction by CAR-T cells. A strategy to enhance the function of CAR-T can be reached by targeting TAAs while treating with anti-PD-1 blocking antibodies in combination (294). The co-application of prostate stem cell antigen (PSCA)-targeted and mucin 1 (MUC1)-targeted CAR-T cells effectively eradicated cancer cells in individuals diagnosed with PSCA and MUC1 positive non-small cell lung cancer (294).

Addressing these barriers is crucial to unlock CAR-cell therapy to its full potential and improve patient outcomes. Research efforts are ongoing to overcome these challenges and advance the application of CAR-T cell therapy in the context of solid malignancies. Enhancing CAR-T cell infiltration into the TME is a critical challenge in solid tumors, including NSCLC. Specific strategies include structurally altering CAR-T cells combined with targeted therapy, radiotherapy, or chemotherapy (295). Chemotherapy has the ability to alter TME and enhance the effectiveness of CAR-T cell therapy. In particular, neoadjuvant therapies, have shown the ability to enhance the effectiveness of GD2 CAR-T cells by decreasing MDSCs in the tumor (295). In a phase I clinical trial, combined therapy involving CAR-T cells along with paclitaxel and cyclophosphamide revealed clinical improvements in 21 of the 28 patients who had previously experienced unsuccessful paclitaxel therapy (296). Murty et al. in 2020 demonstrated in a glioblastoma model that radiotherapy enabled the swift movement of CAR-T cells from the vascular system into the TME, as well as their amplification within the TME, leading to enhanced and prolonged immune responses (297). In order to increase the CAR-T infiltration into the TME, researchers are actively exploring novel approaches. In particular, dual-receptor CAR-T cells have been designed to improve specificity while enhancing T-cell activation and persistence in the TME. These constructs are engineered to recognize two different TAAs simultaneously. By targeting multiple antigens, dual-receptor CAR-T cells can mitigate antigen escape and improve infiltration through stronger, more sustained signaling (298). A second strategy focuses on optimizing the delivery methods for CAR-T cells, directly injecting CAR-T cells into or near the tumor site. Intratumoral delivery reduces systemic exposure, potentially minimizing off-tumor toxicity and enhancing CAR-T cell concentrations at the target site. This strategy has shown promise in preclinical models of glioblastoma and pancreatic cancer and is currently under investigation in lung cancer (299, 300). To further enhance T-cell migration, researchers are modifying CAR-T cells to express chemokine receptors to exploit the chemokine profile of the TME as chemoattractant. For example, expressing CXCR2 allows CAR-T cells to home more effectively to tumors secreting chemokines like CXCL1 and CXCL8. Similar modifications, such as introducing CXCR5 to target CXCL13-rich NSCLC tumors, have shown significant improvements in infiltration and therapeutic outcomes (273, 301, 302). Additionally, combining CAR-T cell therapy with immune checkpoint inhibitors, such as anti-PD-1 or anti-CTLA-4 antibodies, has shown promise in preclinical and early-phase clinical trials for NSCLC. This combination approach can help overcome immune suppression and enhance T-cell function (303, 304). The application and testing of novel strategies through forthcoming clinical trials will be key to assess their real benefits for the broadest population of NSCLC patients.

## Discussion

NSCLC, the predominant form of lung cancer, is a major contributor to cancer-related deaths (4). Over time, however, there have been substantial advancements in the treatment of NSCLC, particularly due to the emergence of targeted therapies that have revolutionized the field toward individualized medical care. Undoubtedly, TKIs have brought about a revolutionary shift in the treatment of individuals with specific genetic mutations (305). Within the realm of targeted therapy, EGFR stands as a quintessential model: activating mutations found in the *EGFR* gene are widespread among a significant portion of individuals afflicted with NSCLC, and can be effectively targeted by TKIs such as erlotinib, gefitinib, and osimertinib, which have markedly improved PSF and OS of many patients (306). Similarly, ALK rearrangements respond well to alectinib and lorlatinib, while MET, BRAF, and ROS1 mutations are targeted by crizotinib, vemurafenib, and dabrafenib (307).

Regrettably, the group of individuals that respond well to TKIs accounts for just 15–20% of all NSCLC patients, and although targeted treatments show promise at first, leading to longer PFS and improved OS, life expectancy is hindered by the development of pharmacological resistance mechanisms for most patients (11). Consequently, the main goal in NSCLC investigations is the design and testing of innovative therapies that impact PFS and OS, taking into account the major role played by the TME in sustaining therapeutic resistance and tumor progression (12). Understanding the tumor TME is key, as it has a substantial impact on how effective these treatments are. Importantly, the presence of neoantigens, TAAs, TSAs, and TILs can define an “inflamed” TME in NSCLC, underscoring its significance in amplifying the immune system’s ability to target tumors by enhancing the effectiveness of neoantigen-based immunotherapies (271). Additionally, the presence of TLS within the TME - organized aggregates of immune cells resembling germinal centers - can further enhance, refine and sustain local and systemic immune responses. TLS have been associated with better prognoses and may play a crucial role in the success of immunotherapies (308).

Immunotherapy has become a cornerstone in NSCLC treatment, with ICIs leading the charge, specifically in those patients without any driver mutations (17). ICIs such as nivolumab and pembrolizumab, which target PD-1, and ipilimumab, targeting CTLA-4, have dramatically improved survival rates for patients with advanced NSCLC (309). The success of these therapies has spurred the development of new ICIs targeting other checkpoint molecules like LAG-3, TIM-3, and TIGIT (310). These novel ICIs aim to overcome resistance mechanisms and further potentiate the immune response against cancer, highlighting the dynamic and expanding landscape of immunotherapy in NSCLC. Yet, a considerable number of patients do not benefit from immunotherapies. In particular, the failure of standard immunotherapy is a frequent issue in the context of a ‘cold’ TME with a lack of neoantigens, diminished T cell numbers, and minimal PD-L1/PD-1 expression (23).

**Figure 1:**
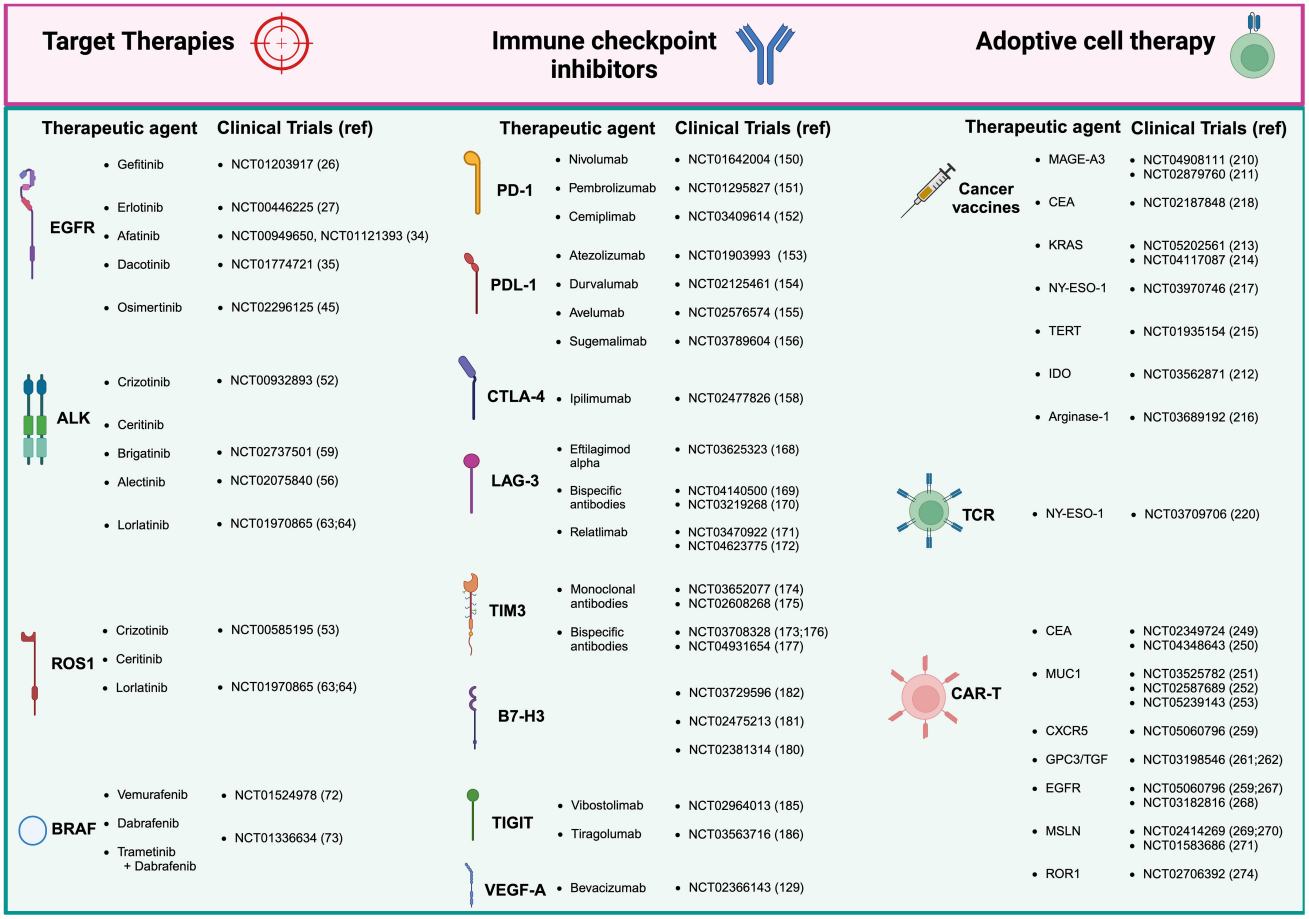
Therapeutic approaches and clinical trials for non-small cell lung cancer (NSCLC). Current treatment of NSCLC range mainly in three areas: tyrosine kinase inhibitors (TKIs), which remain the gold standard for many patients; immune checkpoint inhibitors (ICIs), which have offered exceptional survival benefits to selected patients; adoptive cell therapy, representing the latest frontier in immunotherapy. Adoptive cell therapy divides then into three sub-areas of therapeutic interventions: cancer vaccines, T cell receptor (TCR) based therapy, and the most innovative antigen receptor chimeric (CAR) T cell therapies, which has shown great promise in NSCLC. Created in BioRender. Mazza, M. (2025) https://BioRender.com/o50k263.

**Figure 2:**
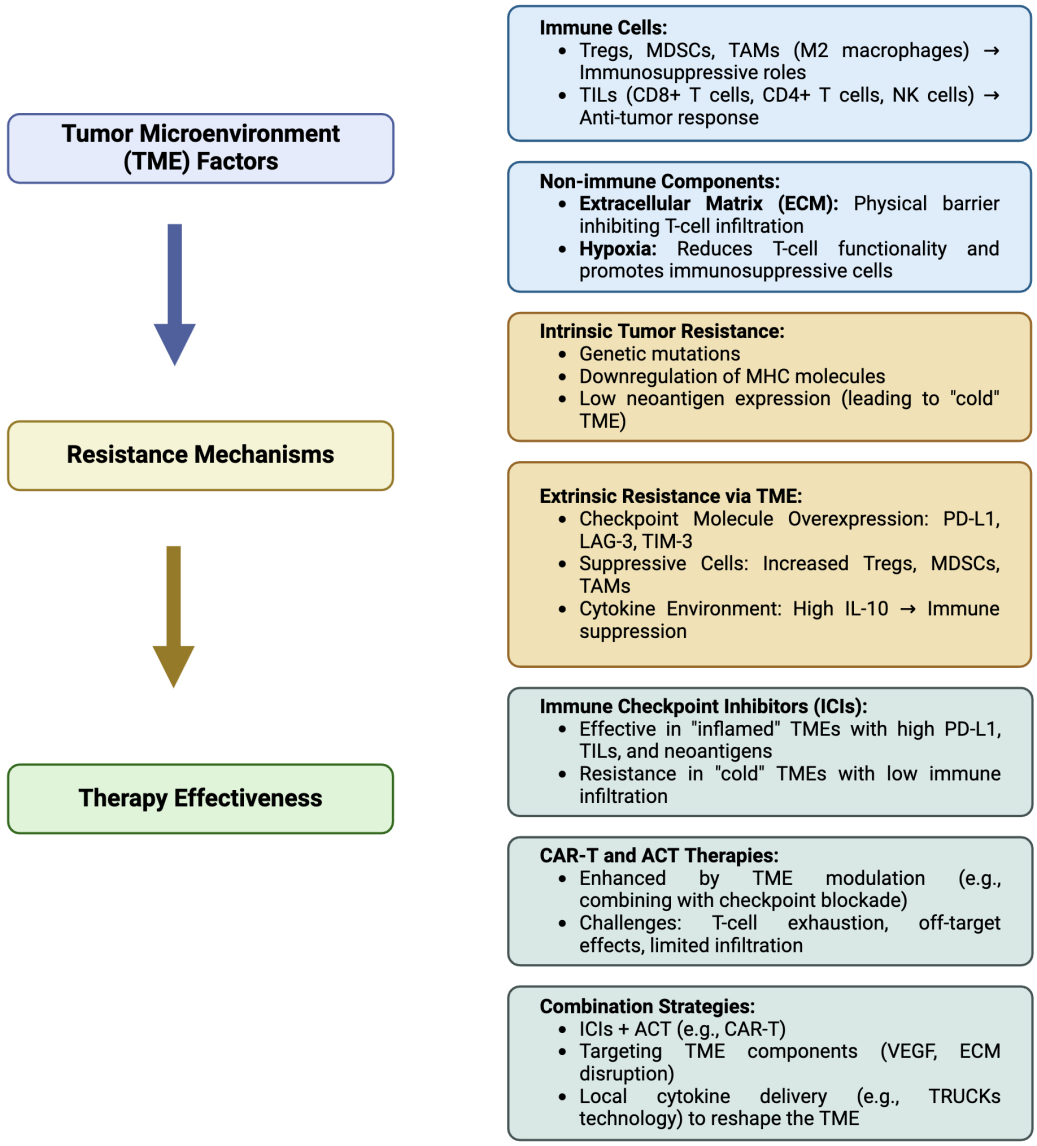
Summarizing flowchart of the relationship between the tumor microenvironment (TME) and associated resistance mechanisms, affecting immunotherapy of non-small cell lung cancer (NSCLC). Tregs, Regulatory T cells; MDSC, Myeloid-derived suppressor cells; TAMs, Tumor-associated macrophages (TAM)-M2; TILs, Tumor-infiltrating lymphocytes; NK, natural killer; MHC, Major histocompatibility complex; PD-L1, Programmed cell death ligand-1; LAG-3, Lymphocyte-activation gene 3; TIM-3, T-cell immunoglobulin, mucin-domain containing-3; IL-10 interleukin-10; ACT, adoptive T cell transfer; CAR-T, Chimeric antigen receptor (CAR)-T cells; VEGF, vascular endothelial growth factor; TRUCKs, T cells redirected for universal cytokine-mediated killing. Created in BioRender. Mazza, M. (2025) https://BioRender.com/m08u912.

The exploration of novel immunotherapeutic approaches is ongoing, with TSA-directed strategies at the forefront. Vaccines targeting TSAs derived from NSCLC common drivers, such as MAGE-A3, CEA, KRAS, NY-ESO-1, and TERT, show great promise in several early trials (311). These new strategies are designed to boost the immune response of the body to target cancer cells in a precise manner and to defeat the immune escape mechanisms triggered by tumors. ACT-based therapies are gaining momentum in the panorama of clinical experimentation. ACT involves infusing patients with T cells able to recognize and attack cancer cells. ACT includes TCR and CAR-T cell therapy, which features genetically modified T cells to express receptors specific to intracellular or membrane associated tumor antigens, respectively. These therapies represent a significant leap forward in the quest for effective and durable cancer treatments (312). CAR-T cell therapy, in particular, has garnered attention for its potential in treating NSCLC. Over successive generations, CAR-T cells have been refined to enhance their efficacy and safety, with modifications aimed at improving persistence, reducing toxicity, and boosting antitumor activity (313). While early results are promising, challenges remain, particularly in managing on-target, off-tumor effects and ensuring sustained responses. Nevertheless, the potential of CAR-T cell therapy to significantly impact NSCLC treatment is undeniable specially *in vivo* transient expression of mRNA in mRNA-engineered T cells, which is a highly promising approach (314). This method eliminates the need to isolate patient cells, by simplifying the production process and allowing for a purely off-the-shelf solution. Another promising format relies on TRUCKs, allowing cytokines and other immunological molecules to be delivered locally at the tumor site to restrain TME immunosuppressive cues and elicit immune responses previously kept in check. Indeed, ACTs are also suited to be combined with ICIs to boost their own activity in difficult contests, promote epitope spreading across APCs and engage preexisting adaptive and innate immunity against tumor cells. In conclusion, understanding the complexities of the TME and leveraging novel treatment strategies that exploit the ability of immune cells to recognize TAAs/TSAs is crucial to improve patient outcomes. Boosted by this primary goal, the treatment landscape for NSCLC is rapidly advancing toward the integration of targeted therapies and innovative immunotherapeutic approaches, while new clinical trials are increasingly looking into how ACT and CAR-T cell therapy can influence NSCLC by targeting diverse surface antigens. In order to guide the reader through the complex scenario of NSCLC therapy options, we provide a brief yet informative summary indicating the reference trials that allowed drug registration in **Figure 1**. As research continues to unravel the intricacies of NSCLC and its microenvironment, the promise of achieving more effective and durable responses and a cure becomes increasingly attainable. In this review, we adeptly merge the literature and highlight the critical hurdles that must be tackled in order to impact effectively on patients’ health, with a specific emphasis on the complexities associated with non-small cell lung cancer, by implementing novel and smarter immunotherapy approaches into clinical practice. In **Figure 2** we have summarized the relationships occurring between the tumor microenvironment (TME) and the associated mechanisms of resistance, indicating the approaches currently under clinical testing to overcome resistance in NSCLC patients.
